# Spanish Versions of the Outcome Rating Scale and the Session Rating Scale: Normative Data, Reliability, and Validity

**DOI:** 10.3389/fpsyg.2021.663791

**Published:** 2021-08-13

**Authors:** Nelson Andrade-González, Irene Rodrigo-Holgado, Jesús Fernández-Rozas, Pablo F. Cáncer, Guillermo Lahera, Alberto Fernández-Liria, Gabriel Rubio, Scott D. Miller

**Affiliations:** ^1^Relational Processes and Psychotherapy Research Group, Faculty of Medicine and Health Sciences, University of Alcalá, Alcalá de Henares, Spain; ^2^Psychotherapy Unit, 12 de Octubre University Hospital, Madrid, Spain; ^3^Faculty of Psychology, Autonomous University of Madrid, Madrid, Spain; ^4^Faculty of Medicine and Health Sciences, University of Alcalá, Alcalá de Henares, Spain; ^5^Biomedical Research Networking Center for Mental Health Network, Ramón y Cajal Institute for Health Research, Madrid, Spain; ^6^Príncipe de Asturias University Hospital, Alcalá de Henares, Spain; ^7^12 de Octubre University Hospital, Madrid, Spain; ^8^12 de Octubre Research Institute, Madrid, Spain; ^9^Faculty of Medicine, Complutense University of Madrid, Madrid, Spain; ^10^The International Center for Clinical Excellence, Chicago, IL, United States

**Keywords:** ORS, SRS, Spain, reliability, validity

## Abstract

Routine outcome monitoring (ROM) uses standardized measures to both track and inform mental health service delivery. Use of ROM has been shown to improve the outcome of psychotherapy when applied to different types of patients. The present research was designed to determine the reliability and validity of the Outcome Rating Scale (ORS) and the Session Rating Scale (SRS) in a sample of Spanish patients. After a controlled process of translation into the Spanish that is spoken and written in Spain (i.e., in Europe, as distinct from, e.g., Latin American Spanish), both measures were completed by patients of an outpatient mental health unit during eight sessions of psychotherapy. Sixty mental health patients filled out the ORS and 59 the SRS. In addition, the ORS was completed by 33 people who constituted the non-clinical sample. The cut-off of the ORS was 24.52 points, and the Reliable Change Index (RCI) was 9.15 points. ORS and SRS scores exhibited excellent internal consistency. The temporal stability of the SRS was adequate. The convergent and discriminant validity of the two measures were adequate. Regarding the factorial validity of the ORS and the SRS, in the third psychotherapy session, confirmatory factor analyses evidenced the existence of a unifactorial model. The predictive validity of SRS was acceptable. The ORS was sensitive to changes in patients’ symptoms. In conclusion, compared to the original English versions of the ORS and SRS measures, the Spanish versions of the measures are also reliable and valid.

## Introduction

Although generally efficacious, psychotherapy is not helpful for all patients. Dropout rates average about 25% for adults and 35% for adolescents and children (Weisz et al., 2005; Swift and Greenberg, 2012). Also, 5–10% of adult patients and 12–20% of child and youth patients’ well-being becomes worse in psychotherapy (Lambert, 2010; Warren et al., 2010). In addition, Hansen et al. (2002) found that the rate of improvement for patients treated in routine practice settings was lower than for patients participating in clinical trials. Although there are patient variables that influence these outcomes, the greatest contribution of therapists to these unsatisfactory outcomes comes from an inadequate therapeutic relationship (Safran et al., 2005). The therapeutic relationship is central to all psychotherapy orientations and modalities and is a major contributor to psychotherapy outcomes (Baldwin et al., 2007).

Accordingly, routine outcome monitoring (ROM) helps to identify when psychotherapy is effective, uncertain, or ineffective and can improve the outcomes of the psychotherapy. ROM – the process of using standardized measures to both track psychotherapy outcomes and the therapeutic alliance to inform mental health service delivery – is the subject of an increasing number of studies and meta-analyses (Lambert et al., 2018). Although a host of potential measures exist, two of the most commonly used and researched are the Outcome Rating Scale (ORS) and Session Rating Scale (SRS) (Prescott et al., 2017). The ORS (Miller et al., 2003) offers information about an individual’s well-being, interpersonal and social functioning, and overall sense of well-being; the SRS (Duncan et al., 2003) provides information about the working alliance as perceived by the patient.

The patient completes the ORS at the beginning of each session and the SRS at the end of the session, usually in the presence of the therapist. To allow this, the clinician must create an atmosphere in which the patient can respond freely to the measures and does not feel judged, perceives that it will improve the care they receive, and knows that the therapist will not be offended if their feedback is negative (Bargmann and Robinson, 2012). Tracking patient scores and feedback alerts the clinician to concerns about progress or the quality of care. Patient ORS scores are compared to normed recovery trajectories (Anker et al., 2009; Duncan, 2012), while scores on the SRS are compared to empirically established cutoff scores (Bargmann and Robinson, 2012; Prescott et al., 2017).

The original validation study of the ORS (Miller et al., 2003) included 435 patients (clinical sample) and 86 people without a mental disorder (non-clinical sample). Before treatment, the mean scores of the patients in the ORS (19.60 points, SD = 8.70) were significantly lower than the scores of the non-clinical sample (28.00 points, SD = 6.80). The reliability and convergent validity of this measure’s scores were obtained in the non-clinical sample on four separate occasions (Ts). Cronbach’s alpha coefficients (internal consistency) of the ORS ranged from 0.87 (T1) to 0.96 (T3 and T4). Regarding temporal stability, the stability coefficients were 0.49, 0.58, and 0.66 (between T1 and T4, between T1 and T3, and between T1 and T2, respectively). Regarding convergent validity, Pearson’s correlation coefficients between the ORS and the Outcome Questionnaire 45.2 (OQ-45; Lambert et al., 1996) fluctuated between 0.53 (T2) and 0.69 (T1). The ORS was significantly sensitive to symptom change experienced by 435 adult patients at the end of the tenth psychotherapy session (Miller et al., 2003).

In the original study of the SRS (Duncan et al., 2003) the reliability and convergent validity of the measure’s scores were obtained from a clinical sample of 70 adult patients in an outpatient setting at different times (Ts) in which the measure was applied. The alpha value of the SRS was 0.88 (obtained over a total of six administrations). Regarding temporal stability, the stability coefficients were 0.70 (between T1 and T2) and 0.64 (overall test–retest reliability). Regarding convergent validity, Pearson’s correlation coefficient between the SRS and the Revised Helping Alliance Questionnaire (HAq-II; Luborsky et al., 1996) was 0.48 (the data from the six administrations were combined). Finally, regarding predictive validity, the correlation coefficient between the second- or third-session SRS scores and the last-session ORS scores was 0.29 (*n* = 100 patients, *p* < 0.01; Duncan et al., 2003).

Subsequently, the ORS and SRS have been validated in a number of studies across different samples of patients treated by therapists of different theoretical orientations [see a review of its psychometric properties in Prescott et al. (2017)]. Furthermore, the psychometric properties of the measures have been assessed for Dutch and Slovak translations (Hafkenscheid et al., 2010; Biescad and Timulak, 2014; Janse et al., 2014). They have also been assessed for two Spanish translations without contrasting if these translations apply to Peninsula Spanish speakers (Moggia et al., 2018, 2020).

The goal of the present research was to get normative, reliability, and validity data from the ORS and the SRS in a sample of Spanish patients. Concerning normative data, it was vital to provide data related to the cut-off for both measures and thus verify their usefulness in clinical practice. This investigation was necessary for three reasons: (1) both measures have been applied to thousands of patients in various countries and have gained popularity in the last decade; (2) it was essential to have data from translations into the Spanish that is spoken and written in Spain (i.e., in Europe, as distinct from, e.g., Latin American Spanish); and (3) the data needed to come from Spanish patients attending a clinical setting within the Spanish public health care system, to extend the use of these measures in the future in the Spanish public healthcare. Consequently, we hypothesize that the scores of the ORS and SRS in Spanish will exhibit adequate normative, reliability, and validity data, similar to those obtained by the original English versions.

## Materials and Methods

### Translation of the ORS and SRS Into Spanish

On July 9, 2013, Dr. Scott D. Miller, founder of the International Center for Clinical Excellence (United States), authorized the first author of this work by email to validate the ORS and the SRS in the Spanish language. Later, the ORS and the SRS were formally translated into Spanish by the Relational Processes and Psychotherapy Research Group of the University of Alcalá (Alcalá de Henares, Madrid, Spain). The process was as follows. First, a professional Spanish translator translated the ORS and the SRS. Next, two Spanish experts in psychotherapy (each of them with more than 20 years of clinical, teaching, and research experience) and two Spanish psychologists examined and approved the translation into Spanish of most of the instructions and items of both versions. Subsequently, the best translation was agreed upon among the translators, the experts, and the professionals. Finally, two linguists from the School of Writing at the University of Alcalá reviewed the two translated measures and reported that they could be understood by most Spanish people.

### Empirical Study

#### Participants

##### Patients

Sixty Spanish outpatients were recruited from the Psychotherapy Unit of the 12 de Octubre University Hospital in Madrid (Spain). Two types of patients were involved: those who received specialized healthcare (*n* = 7) and those who were part of a suicide prevention program (*n* = 53). The sixty patients received seven psychotherapy sessions; 57 of them continued the treatment until the eighth session. All of them gave their consent to participate in this study. The demographic characteristics of the 60 outpatients that formed the clinical sample of this investigation are presented in Table 1. One patient did not provide data on marital status or highest educational level, and one patient did not have data for the SRS in any session. Regarding the diagnoses, the symptoms of each patient were included in one of the following groups of diagnoses: depressive, personality, and anxiety disorders. According to the therapists, 32 of the 60 patients (53.3%) met the DSM-5 diagnostic criteria (American Psychiatric Association, DSM-5 Task Force, 2013) for depressive disorders, 17 (28.4%) met the conditions for personality disorders, and 11 (18.3%) met the criteria for anxiety disorders. Of the total patients, 53 (88.3%) had made a suicide attempt before treatment. Patients who had suffered psychotic symptoms, schizophrenia, or other psychotic disorders, who had suffered a head injury, and/or who had an intellectual disability were excluded from this study. The patients did not receive any incentive to participate in this research.

**TABLE 1:** Demographic characteristics of patients before treatment.

*N*	60	
**Age, years (SD)**	37.2	(13.3)
Range, years	18–70	
**Sex, *n* (%)**		
Male	20	(33.3%)
Female	40	(66.7%)
**Marital status, *n* (%)**		
Single	27	(45.0%)
Separated/divorced	9	(15.0%)
Married/partnered	23	(38.3%)
**Highest educational level, *n* (%)**		
No studies	2	(3.3%)
Elementary	17	(28.3%)
High school	30	(50.0%)
University	10	(16.7%)
**Employment, *n* (%)**		
Unemployed	18	(30.0%)
Student	5	(8.3%)
Employed	33	(55.0%)
Self-employed	1	(1.7%)
Retired	3	(5.0%)

##### Therapists

The clinician sample included two men (18.2%) and nine women (81.8%) aged between 25.5 and 59.2 years (*M* = 32.7 years; SD = 9.4). Of the 11 therapists, one was a clinical psychologist (9.1%) and 10 resident psychologists (90.9%). As for their theoretical orientation, nine therapists were integrative and two psychodynamic. The integrative therapists tailored the treatment to the needs of each patient and used techniques and strategies from different theoretical orientations. The clinical experience of all therapists ranged between 1 and 35 years (*M* = 4.7 years; SD = 10.0).

##### Non-clinical Sample

A group of 33 Spanish people recruited by snowball sampling constituted the non-clinical sample. Their ages ranged from 20 to 59 years (*M* = 32.0 years; SD = 10.0); 51.5% were male. Twenty-nine of these participants were single, two were separated/divorced, and two were married/partnered. As for their highest level of education, twenty-nine of them had a university degree, whereas the other four had secondary studies.

#### Treatment

All patients received 1-h individual psychotherapy sessions with 51.7% also receiving psychopharmacological treatment. The psychotherapy sessions occurred weekly or biweekly. Fifty-two patients were treated with integrative psychotherapy and eight with psychodynamic psychotherapy; patients were not randomized to these treatment conditions. In the present study, the scores of patients and therapists on the different measures were computed until the end of the eighth psychotherapy session.

#### Measures

##### Spanish-Language Outcome Rating Scale (ORS)

The ORS (Miller et al., 2003) is a self-report measure that provides information about four dimensions of well-being: individual well-being, interpersonal functioning (in their closest relationships), social functioning (outside the home/community), and a general sense of well-being. The measure has four 10 cm visual analog scales (items) each representing one of the dimensions listed above. The patient marks the scale nearest to whichever “pole” describes their experience best where marks to the left represent lower marks and to the right on the scale represent higher marks. Scores are obtained by placing a ruler on the each scale to find the corresponding value of each mark. Each scale has a maximum value of 10 points (for example, if the patient’s mark on the scale measures 6.5 cm from the left pole, the score for that scale is 6.5). The scores on each of the four scales are added together for a total ORS score ranging from 0 to 40 points; a higher score indicates better functioning of the patient. The Spanish ORS can be downloaded from the following website: http://tiny.cc/ankujz.

##### Spanish-Language Session Rating Scale (SRS)

The SRS (Duncan et al., 2003) is a 4-item visual analog self-report measure of the working alliance as perceived by the patient. It is based on the components of the working alliance as defined by Bordin (1979). Specifically, the SRS measures the relational bond between the patient and therapist, agreement on goals, agreement on tasks, and the patient’s general perception of the alliance. The structure of the SRS, its mode of use, and the range of scores are identical to those of the ORS. A higher SRS score indicates a better alliance as perceived by the patient. The Spanish SRS can be downloaded from the following website: http://tiny.cc/ankujz.

##### Spanish-Language Clinical Outcomes in Routine Evaluation – Outcome Measure (CORE-OM)

The CORE-OM [original version by Barkham et al. (1998) and Evans et al. (2000); Spanish version by Trujillo et al. (2016)] is a self-report scale that evaluates four aspects of the adult patient: subjective well-being, symptomatology (mainly anxious and depressive), functioning (general, interpersonal, and social), and risk (to oneself and others) using 34 items, each with five response options ranging from 0 (*not at all*) to 4 (*most or all of the time*). The scoring range for the overall CORE-OM is 0*–*136 points; the higher the score on all of these four domains, the greater the symptoms of the patient. In the present study, the internal consistency before treatment, after the third psychotherapy session, and after the eighth session was excellent (Cronbach’s alphas = 0.92, 0.95, and 0.95, respectively).

##### Spanish-Language Working Alliance Inventory, Short Form for Patients (WAI-S-P)

The WAI-S-P [original version by Tracey and Kokotovic (1989); Spanish version by Andrade-González and Fernández-Liria (2016)] is a self-report that measures the alliance perceived by the patient according to Bordin’s (1979) model; for this, it has 12 items, each with seven response options ranging from 1 (*never*) to 7 (*always*). The scoring range of the overall WAI-S-P is 12*–*84 points; the higher the score, the greater the alliance. In the present study, the internal consistency after the third and eighth psychotherapy sessions was excellent (Cronbach’s alphas = 0.88 and 0.91, respectively).

##### Spanish-Language Working Alliance Inventory, Short Form for Therapists (WAI-S-T)

The WAI-S-T [original version by Tracey and Kokotovic (1989); Spanish version by Andrade-González and Fernández-Liria (2016)] is a self-report that measures the alliance as perceived by the therapist according to Bordin’s (1979) model. The WAI-S-T has the same number of items, the same response options, and the same scoring range as the WAI-S-P; again, the higher the score, the greater the alliance. In the present study, its internal consistency after the third and eighth psychotherapy sessions was excellent (Cronbach’s alphas = 0.90 and 0.95, respectively).

#### Procedure and Data Analysis

The Ethics Committee of the 12 de Octubre University Hospital in Madrid (Spain) approved the study. Before treatment, patients signed a written consent form to participate in this research and completed a socio-demographic fact sheet. At the same time, separately, their therapists completed another socio-demographic fact sheet. Before each of the eight psychotherapy sessions, patients completed the ORS; at the end of these sessions, the patients filled in the SRS. The clinicians introduced these two measures in an atmosphere that favored patient feedback and discussed the results with them. Before treatment and after the third and eighth psychotherapy sessions, patients completed the CORE-OM; in addition, at the end of the third and the eighth sessions, the patients completed the WAI-S-P and the therapists the WAI-S-T. The patients were unaware of the therapists’ responses in the WAI-S-T, while the therapists were not aware of the patients’ responses in the CORE-OM and WAI-S-P. The people in the non-clinical sample completed the ORS eight times; the mean interval between applications was 7.00 days (SD = 0.00).

IBM SPSS Statistics, version 20.0, and *OpenMx* (Neale et al., 2016), software packages were used to analyze the data. The corrected item–total correlations of the ORS and SRS items were obtained by correlating the score of each item with the total score of their respective scale minus that item. To assess the differences between the mean scores of the clinical and non-clinical samples in the ORS, a *t*-test for two independent samples was used. To examine the evolution of the scores of the patients in the ORS and the SRS during the eight sessions of psychotherapy, a mixed model with a repeated-measures factor was used. To establish the existence of clinically significant change in a patient on the ORS, Jacobson and Truax’s (1991) method was used. First, the cut-off (C) was calculated, before the psychotherapeutic treatment; the formula used was *C* = [(*SD_*f*_ ⋅ M_*c*_*) + (*SD_*c*_ ⋅ M_*f*_*)] / (*SD_*f*_ + SD_*c*_*) where *M*_*f*_ and *SD*_*f*_ are the mean and the standard deviation respectively of the functional sample (the non-clinical sample) and *M*_*c*_ and *SD*_*c*_ the mean and standard deviation respectively of the clinical sample. Next, the Reliable Change Index (RCI) was calculated before treatment, multiplying the standard error of difference between two scores (*S*_*diff*_) by the *z* value of the requisite significance level (1.96, *p* < 0.05). The formula for calculating the standard error was *S*_*diff* =_ $\sqrt{2{\left(s_{e}\right)}^{2}}$ = $\sqrt{2{\left(s_{x}\sqrt{\left(1-r_{xx}\right)}\right)}^{2}},$where *s*_*x*_ is the standard deviation of the ORS scores in the clinical sample and *r*_*xx*_ the internal consistency of this measure in this sample. To estimate reliability in terms of internal consistency and temporal stability of ORS and SRS scores, Cronbach’s alpha coefficient and the stability coefficient were respectively used. The Pearson correlation coefficient was utilized to assess the convergent/discriminant validity of the scores of both measures. Regarding factorial validity, before performing the confirmatory factor analyses, we conducted a Monte Carlo simulation with the *mxPowerSearch()* function in *OpenMx* to examine how many participants were needed to obtain the estimated factor loadings for a power of 0.80. Minimum sample size requirements for the ORS were 51 in both the third and eight sessions, and for the SRS, they were 63 for the third session and 22 for the eight session. Because the sample size used was close to the minimum requirements, we used bootstrapping for more accurate standard errors. The single-factor solutions for the ORS and the SRS were tested separately by confirmatory factor analysis (CFA) with maximum likelihood estimation, according to the single-factor model proposed by Quirk et al. (2013) for the Group SRS. In order to evaluate model fit, the chi-squared statistic (χ^2^), Comparative Fit Index (CFI), Tucker–Lewis Index (TLI), Root–Mean–Square Error of Approximation (RMSEA), and Standardized Root–Mean–Square Residual (SRMR) were computed. To evaluate the sensitivity to change of the ORS, a paired *t-*test was used. Finally, regression analyses were utilized to estimate the predictive validity of the SRS scores (after the third psychotherapy session). Missing data were removed from the analyses by pairwise deletion.

## Results

### Item Analyses and Normative Data

Patients had a higher mean age than participants in the non-clinical sample (*p* < 0.01). In the clinical and non-clinical samples, the proportion of males and women was similar (*p* = 0.07); however, both samples differed in marital status and educational level (*p* < 0.01). Meanwhile, the corrected item–total correlations of all items of the Spanish ORS and SRS in the eight sessions of psychotherapy were respectively >0.64 and >0.57. The mean scores on the ORS and the SRS are presented in Table 2. The mean scores of the clinical sample on the ORS were significantly lower than those of the non-clinical sample until the fourth psychotherapy session (*p* < 0.05). The mean scores of the patients on the ORS increased significantly after eight sessions (*F*_7,410_ = 35.27, *p* < 0.01); the same occurred with the SRS (*F*_7,403_ = 7.34, *p* < 0.01). The cut-off that a patient had to overcome to move from a dysfunctional to a functional distribution in the ORS was 24.52 points. The RCI was 9.15 points. The percentages of patients who obtained less than 36 points on the SRS were the following: first session: 45.77%; second session: 37.29%; third session: 28.82%; fourth session: 25.43%; fifth session: 25.43%; sixth session: 20.34%; seventh session: 15.26%; and eighth session: 21.43%.

**TABLE 2:** Mean scores, standard deviations, alpha coefficients, and stability coefficients of the Spanish versions of the Outcome Rating Scale (ORS) and the Session Rating Scale (SRS).

Outcome Rating Scale (ORS)																							

Clinical sample																							

Session 1 (*n* = 60)			Session 2 (*n* = 60)			Session 3 (*n* = 60)			Session 4 (*n* = 60)			Session 5 (*n* = 60)			Session 6 (*n* = 60)			Session 7 (*n* = 60)			Session 8 (*n* = 57)		
							
*M*	SD	α	*M*	SD	α	*M*	SD	α	*M*	SD	α	*M*	SD	α	*M*	SD	α	*M*	SD	α	*M*	SD	α
16.26	9.53	0.88	20.16	9.73	0.89	21.90	10.41	0.93	25.21	10.70	0.94	27.64	9.57	0.92	29.57	9.86	0.95	29.25	9.25	0.93	30.96	9.50	0.93
			*r*_*x1x2*_ = 0.60**			*r*_*x1x3*_ = 0.39**			*r*_*x1x4*_ = 0.35**			*r*_*x1x5*_ = 0.30*			*r*_*x1x6*_ = 0.17			*r*_*x1x7*_ = 0.21			*r*_*x1x8*_ = 0.27*		
**Non-clinical sample**																							

**Time 1 (*n* = 33)**			**Time 2 (*n* = 33)**			**Time 3 (*n* = 33)**			**Time 4 (*n* = 33)**			**Time 5 (*n* = 33)**			**Time 6 (*n* = 33)**			**Time 7 (*n* = 33)**			**Time 8 (*n* = 33)**		
							
***M***	**SD**	**α**	***M***	**SD**	**α**	***M***	**SD**	**α**	***M***	**SD**	**α**	***M***	**SD**	**α**	***M***	**SD**	**α**	***M***	**SD**	**α**	***M***	**SD**	**α**

29.36	5.57	0.90	28.39	4.29	0.74	30.64	4.30	0.89	30.60	4.80	0.84	30.45	4.52	0.85	30.36	4.61	0.87	30.50	4.05	0.77	31.10	3.85	0.85
			*r*_*x1x2*_ = 0.61**			*r*_*x1x3*_ = 0.26			*r*_*x1x4*_ = 0.42*			*r*_*x1x5*_ = 0.42*			*r*_*x1x6*_ = 0.13			*r*_*x1x7*_ = −0.03			*r*_*x1x8*_ = 0.36*		

**Session Rating Scale (SRS)**																							

**Clinical sample**																							

**Session 1 (*n* = 59)**			**Session 2 (*n* = 59)**			**Session 3 (*n* = 59)**			**Session 4 (*n* = 59)**			**Session 5 (*n* = 59)**			**Session 6 (*n* = 59)**			**Session 7 (*n* = 59)**			**Session 8 (*n* = 56)**		
							
***M***	**SD**	**α**	***M***	**SD**	**α**	***M***	**SD**	**α**	***M***	**SD**	**α**	***M***	**SD**	**α**	***M***	**SD**	**α**	***M***	**SD**	**α**	***M***	**SD**	**α**

34.14	6.81	0.90	35.22	6.37	0.87	36.28	4.89	0.88	36.19	5.95	0.96	36.33	5.09	0.91	37.12	4.48	0.90	37.49	3.86	0.95	37.66	4.01	0.95
			*r*_*x1x2*_ = 0.70**			*r*_*x1x3*_ = 0.71**			*r*_*x1x4*_ = 0.58**			*r*_*x1x5*_ = 0.60**			*r*_*x1x6*_ = 0.56**			*r*_*x1x7*_ = 0.62**			*r*_*x1x8*_ = 0.61**		

ORS, Spanish-Language (SL) Outcome Rating Scale; SRS, SL Session Rating Scale. **p < 0.01 (two-tailed); *p < 0.05 (two-tailed).

### Reliability

The Cronbach’s alpha coefficients and stability coefficients of the Spanish ORS and SRS are in Table 2. The medians of the alpha coefficients of the ORS in the clinical and non-clinical samples were 0.93 (SD = 0.02) and 0.85 (SD = 0.05), respectively, while the median of the alpha coefficients of the SRS was 0.90 (SD = 0.03). In addition, the medians of the stability coefficients of the ORS in the clinical and non-clinical samples were 0.30 (SD = 0.14) and 0.36 (SD = 0.21), respectively, while the median of the stability coefficients of the SRS was 0.61 (SD = 0.05).

### Validity

Regarding convergent/discriminant validity, the Spanish ORS scores were negatively correlated with CORE-OM scores before treatment (*r* = −0.41, *p* < 0.01), in the third psychotherapy session (*r* = −0.41, *p* < 0.01), and in the eighth psychotherapy session (*r* = −0.71, *p* < 0.01). Also, the ORS scores (at the third session) were not significantly correlated with patients’ family history of mental health (treated as a dichotomic variable: NO = 0, YES = 1). On the other hand, the Spanish SRS scores in the third and the eighth sessions were positively correlated with the WAI-S-P scores (*r* = 0.64 and *r* = 0.59, respectively; *p* < 0.01) and with the WAI-S-T scores (*r* = 0.35 and *r* = 0.37, respectively; *p* < 0.01). Also, the SRS scores (at the third session) were not significantly correlated with CORE-OM and ORS scores (before treatment).

Regarding factorial validity, single-factor confirmatory models were fit to the data from the ORS and SRS measures in the third and eighth psychotherapy sessions. The factor loadings for each measure were very high across sessions (see Table 3), meaning that all the responses to the items were strongly correlated with the factor. Standard errors of these estimates were slightly high (see Table 3), indicating similar levels of accuracy for all the factor loadings. In the third session, a single-factor model in the ORS (*N* = 60) showed an excellent fit to the data in all indices, $\chi_{2}^{2}$ = 0.59 (*p* = 0.74), CFI = 1.00, TLI = 1.02, RMSEA < 0.01, and SRMR = 0.01; however, in the eighth session, this model in the ORS (*N* = 57) did not fit the data well, $\chi_{2}^{2}$ = 31.31 (*p* < 0.01), CFI = 0.88, TLI = 0.65, RMSEA = 0.51, and SRMR = 0.09. After the third psychotherapy session, a single-factor model in the SRS (*N* = 59) showed an excellent fit to the data, $\chi_{2}^{2}$ = 0.09 (*p* = 0.95), CFI = 1.00, TLI = 1.03, RMSEA < 0.01, and SRMR < 0.01; and after the eighth session, this model in the SRS (*N* = 56) was acceptably fitted to the data, $\chi_{2}^{2}$ = 11.11 (*p* < 0.01), CFI = 0.97, TLI = 0.91, RMSEA = 0.29, and SRMR = 0.02.

**TABLE 3:** Factor loadings and standard errors of the Spanish versions of the Outcome Rating Scale (ORS) and the Session Rating Scale (SRS).

	Outcome Rating Scale (ORS)				Session Rating Scale (SRS)			
	Third session		Eighth session		Third session		Eighth session	
	λ	bSE	λ	bSE	λ	bSE	λ	bSE
Item 1	0.95	0.02	0.97	0.01	0.94	0.04	0.91	0.05
Item 2	0.82	0.05	0.79	0.07	0.67	0.21	0.81	0.10
Item 3	0.80	0.05	0.70	0.09	0.90	0.08	0.96	0.02
Item 4	0.94	0.03	0.98	0.01	0.84	0.08	0.99	0.02

*ORS, Spanish-Language (SL) Outcome Rating Scale; SRS, SL Session Rating Scale; λ = factor loadings; bSE = standard errors calculated with bootstrapping.*

Regarding the predictive validity of the Spanish version of the SRS, the regression analyses indicated that patient SRS scores at the third psychotherapy session significantly predicted (1) the ORS scores in the sixth session (*r* = 0.35, *p* = 0.00, *B* = 0.703, and bootstrap CI = [0.28, 1.32]), (2) the ORS scores in the seventh session (*r* = 0.25, *p* = 0.02, *B* = 0.481, and bootstrap CI = [0.04, 0.94]), and (3) the CORE-OM scores in the eighth session (*r* = −0.25, *p* = 0.03, *B* = −0.117, and bootstrap CI = [−0.21, 0.047]). The patient SRS scores after the third session did not correlate significantly with their ORS scores in the eighth session (*r* = 0.20, *p* = 0.053, *B* = 0.382, and bootstrap CI = [−0.096, 0.857]).

### Sensitivity to Change

The Spanish ORS was sensitive to change in symptoms experienced by the patients after eight sessions of psychotherapy (ORS 1st – 8th session: mean = −3.70, bootstrap CI = [−3, −4.5], *t* = −9.67, *p* = 0.00, and δ = 1.31). This did not happen in the non-clinical sample (ORS 1st – 8th administration: mean = −0.43, bootstrap CI = [−0.92, 0.004], *t* = −1.80, *p* = 0.08, and δ = 0.32). These analyses achieved a power of 1.0 and 0.78 for the clinical and non-clinical samples, respectively.

Table 4 exhibits a comparison of the main results obtained by the English and the Spanish versions of the ORS and the SRS.

**TABLE 4:** Comparison of the main results of the English and the Spanish versions of the Outcome Rating Scale (ORS) and the Session Rating Scale (SRS).

	American version (English)	American version (Spanish)	Spanish version	
	**Outcome Rating Scale (ORS)**			
Mean scores (CS)	19.60^a^	18.18^b^	16.26^a^	
Mean scores (NCS)	28.00^a^	n/a	29.36^a^	
Cut-off (CS)	25.00	n/a	24.52	
RCI (CS)	5.00	n/a	9.15	
Reliability				
Internal consistency (NCS)	0.93^c^	0.96^c^	0.85^c^	
Temporal stability (NCS)	0.66**^d^	n/a	0.61**^d^	
Convergent validity	0.56**^e^	−0.70*^b^	−0.41**^f,g^	
Sensitivity to symptomatic change (CS)	Yes	Yes	Yes	
	**Session Rating Scale (SRS)**			
Cut-off (CS)	36.00	35.50	36.00	
Reliability				
Internal consistency (CS)	0.88^c^	0.94^c^	0.90^c^	
Temporal stability (CS)	0.64**^c^	0.64*^d^	0.61^h^	
Convergent validity (CS)	0.48**^i^	0.60*^c^	0.64**^j^	
Predictive validity (CS)	0.29**^k^	−0.20*^l^	0.20^m^	−0.25*^n^

ORS, Spanish-Language (SL) Outcome Rating Scale; SRS, SL Session Rating Scale; CS, clinical sample; ^a^before treatment; ^b^at first session; NCS, non-clinical sample; n/a, not available; RCI, Reliable Change Index; ^c^all administrations; ^d^between the first and second administration; ^e^correlation between the ORS total score and Outcome Questionnaire-45.2 total score on the non–clinical sample (fourth administration); ^f^correlation between the SL ORS total score and SL Clinical Outcomes in Routine Evaluation–Outcome Measure (CORE–OM) total score on the clinical sample (third session); ^g^the negative correlation between the SL ORS and the SL CORE–OM occurred because better patient functioning is indicated by high scores in the ORS and low scores in the CORE–OM; ^h^median of the stability coefficients; ^i^correlation between the SRS total score and Revised Helping Alliance Questionnaire total score (all administrations); ^j^correlation between the SL SRS total score and SL Working Alliance Inventory total score, short form for patients (third session); ^k^correlation between the second or third session SRS scores and the final session ORS scores; ^l^correlation between the third session SRS scores and the last session CORE-OM scores; ^m^correlation between the third session SL SRS scores and the eighth session ORS scores; ^n^correlation between the third session SL SRS scores and the eighth session CORE-OM scores.**p < 0.01 (two-tailed); *p < 0.05 (two-tailed).

## Discussion

In the present study, the Spanish ORS and SRS proved to be reliable and valid. The corrected item-total correlations of all items of the Spanish ORS and SRS for the eight sessions of psychotherapy reveal that these elements correlated quite well with the totals of their respective scales. Regarding the mean ORS scores, the lowest values obtained from the clinical Spanish sample compared to the American sample (see Table 4), may be due to the greater clinical severity of the Spanish sample (more than 88% of the Spanish patients had attempted suicide before the start of psychotherapy). The mean ORS scores in the Spanish non-clinical sample at the beginning of the study are comparable with those of the American sample. In both countries, the differences between the scores of the clinical sample (before treatment) and those of the non-clinical sample were statistically significant. In Spain, the absence of differences between the clinical and non-clinical samples from the fifth administration onward of the ORS evidenced that the scores of patients, who received treatment, approximated those of people who did not suffer from any disorder. It’s important to mention that the significant increase in patient scores on the ORS seen here has also been found in American patients (Reese et al., 2009); likewise, the increase in the Spanish SRS scores is in line with what was found in a sample of Dutch patients (Janse et al., 2017). The cut-off and the RCI of the American and Spanish ORS are in Table 4. As for the RCI, the Spanish patients need to achieve a greater change on the ORS than American patients in order for this change to be reliable. In sum, if an adult patient begins a psychotherapy treatment with less than 24.52 points on the Spanish ORS, earns 9 or more points on this scale over the course of the treatment, and at the end of the treatment crosses the ORS cut-off, then this patient will have achieved a clinically significant change. Finally, from the third psychotherapy session onward, the mean scores of the Spanish SRS were higher than the cut-off of the American SRS (36 points; Reese et al., 2009); also, in sessions 4, 5, 6, 7, and 8 the percentage of patients with less than 36 points was 25.43% or less (American data indicate that 24% of cases fall below 36 points; Miller and Duncan, 2004). Consequently, it is suggested that the cut-off of the Spanish SRS be the same as that established in the American sample, 36 points.

Regarding reliability, the internal consistency of the Spanish ORS and SRS scores was excellent; these results show a high degree of covariance between the items of each measure and are similar to those found in the studies that first developed and presented the ORS (Miller et al., 2003) and the SRS (Duncan et al., 2003). As for the temporal stability of the ORS scores, the lower stability of the ORS in the clinical sample compared to the non-clinical sample may have been due to the ability of this measure to detect change in the symptoms of the patients. In the non-clinical sample, a detailed analysis reveals that the American and the Spanish ORS stability coefficients obtained between the first and second application were similar (see Table 4). Further, the temporal stability of the SRS scores was adequate; the median of the stability coefficients of the Spanish version of the SRS was similar to the overall test–retest reliability of the American version (see Table 4), and the stability coefficient between the first and second psychotherapy sessions was the same as that found by the authors of the American SRS (Duncan et al., 2003). Lastly, it is worth mention that the reliability values obtained by the Spanish ORS and SRS are similar to values found in the Slovak translations (Biescad and Timulak, 2014), Dutch (Hafkenscheid et al., 2010; Janse et al., 2014), and those found in the Spanish translations conducted by the team of the original American authors (Moggia et al., 2018, 2020).

Regarding construct validity, the convergent validity of the Spanish ORS scores was adequate in the third psychotherapy session and excellent in the eighth session. The negative correlations between the ORS and the CORE-OM occurred because better patient functioning is indicated by high scores in the ORS and low scores in the CORE-OM. These results are not comparable with those for the ORS in English, since the American convergent validity data were obtained in a non-clinical sample (Miller et al., 2003). However, the correlation between the Spanish ORS and the Spanish CORE-OM (before treatment) was not as strong as that found by Janse et al. (2014) between the Dutch ORS and the Dutch OQ-45 (at intake; *r* = −0.62) nor of that found by Moggia et al. (2018) between the Spanish ORS translated by the original authors and the Spanish CORE-OM (at first session; *r*_*s*_ = −0.70). In any case, the ORS and CORE-OM in Spanish are related to some extent, since they correlated significantly on three occasions, with an especially high correlation between them in the eighth psychotherapy session. Regarding the correlations between SRS scores and WAI-S scores for patients, the convergent validity of the SRS was excellent after the third and eighth psychotherapy sessions. Also, these correlations were similar to those found by Moggia et al. (2020) and higher than those found by Janse et al. (2014) in the Netherlands and by Duncan et al. (2003) in the SRS creation study (see Table 4). The fact that in Spain the correlations were not high between the perceived alliance scores of patients on the SRS and those of the therapists on the WAI-S-T is in line with the moderate results of the studies that have examined patients’ and clinicians’ perceptions of the alliance (Tryon et al., 2007; Andrade-González et al., 2017). On the other hand, the discriminant validity of the Spanish ORS and SRS was adequate, since, as expected, their scores diverged from the scores of measures that evaluate other constructs and from a variable related neither to the patients’ symptomatology nor to the working alliance. Regarding the factorial validity of the ORS and SRS, in the third psychotherapy session, the confirmatory factor analyses provided evidence in favor of a single-factor model. The fact that in the eighth session this model did not fit the data well for the ORS, while it fit acceptably for the SRS, is probably due to lower variability of the scores of both instruments at the time of treatment.

The predictive validity of the Spanish SRS was acceptable because the correlations between the scores on this measure obtained after the third psychotherapy session and those of the ORS and CORE-OM obtained later in the treatment were in the expected direction. In fact, similar to the study by Moggia et al. (2020), Spanish SRS scores predicted patients’ scores on the CORE-OM (see Table 4). However, unlike what happened for the SRS in English (see Table 4), the Spanish SRS scores (at the third session) did not correlate significantly with those of the ORS (at the eighth session). This result was probably due to the relatively small size of the patient sample. The predictive validity of the SRS in the Spanish context will probably improve if the next investigations employ a greater number of patients and make it possible for clinicians to know the mean SRS scores obtained in this study.

The Spanish ORS was sensitive to change in the symptoms of the patients and remained stable in the non-clinical sample. This result is consistent with that found in the American ORS validation study (Miller et al., 2003), and the Dutch (Janse et al., 2014) and Spanish translation of the original authors (Moggia et al., 2018), suggesting that the ORS achieves its purpose: to monitor the patient’s progress in four focal areas of functional well-being.

This work has the following limitations that are important to acknowledge. First, the sample size of the clinical and non-clinical groups was small, which limits the statistical power of this research. Second, there was a clear imbalance in the total number of patients treated, since more than 88% of them had specific characteristics (had made a previous suicide attempt), which makes it difficult to generalize the results to other patient samples that can provide feedback in psychotherapy. Third, it cannot be guaranteed that symptom change in the patients who completed the Spanish ORS is due only to the psychotherapy received (some uncontrolled patient, therapist, and interaction variables, and even a series of extra–therapeutic factors, may also have been influential). Fourth, in the eighth session of psychotherapy, no data could be obtained from the three patients who finished the treatment in the previous session. Fifth, the ORS and the SRS are self-reports that do not measure the degree of social desirability of patient responses. Despite this, both ultra-brief measures exhibited appropriate psychometric properties. In addition, the design of this study made it possible to monitor the symptoms and alliance throughout eight sessions and have an adjusted view of the course of both variables. Finally, a remarkable aspect of our findings is that these measures were applied many times to a sample made up of patients with prevalent disorders who received treatment in the Spanish public health care system (the ORS was applied 477 times to patients and 264 times to people in the non-clinical sample; the SRS was administered 469 times to the patients).

In conclusion, the Spanish versions of the ORS and the SRS have normative, reliability, and validity data comparable to those of the original American versions. Future studies should provide more data related to the psychometric properties of these Spanish versions (mainly on their factorial validity and the predictive validity of SRS) and develop computer procedures that facilitate their use in clinical practice.

## Data Availability Statement

The original contributions presented in the study are included in the article/supplementary material, further inquiries can be directed to the corresponding author.

## Ethics Statement

This study involved human participants and was reviewed and approved by the Ethics Committee of the 12 de Octubre University Hospital in Madrid (Spain). The patients provided their written informed consent to participate in this study.

## Author Contributions

NA-G and JF-R conceived and designed the study. IR-H and JF-R performed the material preparation and data collection. NA-G wrote the manuscript. NA-G and PC performed the data analysis with input from all authors. GL, AF-L, GR, and SM contributed to the interpretation of the results. All authors contributed significantly to the discussion of the findings and approved the final manuscript.

## Conflict of Interest

The authors declare that the research was conducted in the absence of any commercial or financial relationships that could be construed as a potential conflict of interest.

## Publisher’s Note

All claims expressed in this article are solely those of the authors and do not necessarily represent those of their affiliated organizations, or those of the publisher, the editors and the reviewers. Any product that may be evaluated in this article, or claim that may be made by its manufacturer, is not guaranteed or endorsed by the publisher.
